# Biting the Nails

**Published:** 1896-03

**Authors:** 


### Biting the Nails.

Dr. Bertillon, as the result of an extensive inquiry, confirms his previously expressed opinion that onychophagia and similar habits are generally associated with degeneracy. The frequency of onychophagia varies greatly in different institutions. In some, two or three out of every ten children are addicted to biting their nails. A careful examination invariably reveals signs of degeneracy. The children are usually less healthy in appearance than others, presenting deformities of the skull and anomalies of the teeth and ears. In such subjects the teachers notice a marked antipathy to physical exercises and games requiring effort. They write poorly, and show marked inferiority in respect to manual dexterity. They are slow to learn; they are incapable of continuous application; in fact, they always exhibit an inferiority in some direction or other. The disciplinary measures usually resorted to, to correct bad habits are powerless in this: in the majority of cases only hypnotic suggestion seems to be capable of effecting a cure. The habit of biting the nails sometimes persist until late in life.—*Med. Fortnightly.*

